# Thirty-day hospital readmission and its determinants among patients with severe community-acquired pneumonia: a prospective cross-sectional study in Northwest Ethiopia

**DOI:** 10.1186/s12879-026-13064-5

**Published:** 2026-03-17

**Authors:** Masho Tigabe Tekle, Abdisa Gemedi Jara, Faisel Dula Sema

**Affiliations:** Department of Clinical Pharmacy, School of Pharmacy, College of Medicine and Health Sciences, University of Gondar, Gondar, Ethiopia

**Keywords:** Severe community acquired pneumonia, 30-day readmission, Associated factors

## Abstract

**Background:**

Hospital readmissions are a frequent complication of community-acquired pneumonia (CAP), resulting in significant clinical and economic burdens. In Ethiopia, data on 30-day readmissions and related factors are lacking. This study aimed to determine the prevalence and factors associated with 30-day all-cause readmission after severe CAP hospitalization at the University of Gondar Comprehensive Specialized Hospital (UOGCSH).

**Methods:**

A cross-sectional study was conducted From July 1, 2023, to June 30, 2024, among 177 patients with severe CAP who had been discharged alive from the UOGCSH. Patients were selected using a consecutive sampling technique, and severe CAP was defined according to the Infectious Diseases Society of America (IDSA) criteria. Multivariable binary logistic regression was used to identify factors associated with 30-day all-cause hospital readmission, and the results were reported with a 95% CI. Statistical significance was set at *p* < 0.05.

**Results:**

The prevalence of 30-day all-cause hospital readmission was 29.9% (95% CI: 23.3 to 37.3); of those, the majority (71.7%) were readmitted within two weeks. Chronic obstructive pulmonary disease (AOR = 4.51; 95% CI: 1.19 - 17.15), fever (AOR = 3.12; 95% CI: 1.26 - 7.73), admission with ≥3 comorbidities (AOR = 2.68; 95% CI: 1.11 - 6.50), presence of ≥ 1 clinical instability factor at discharge (AOR = 2.54; 95% CI: 1.06 - 6.13), complications of severe CAP, including parapneumonic effusion (AOR = 3.13; 95% CI: 1.26 - 7.77) and respiratory failure (AOR = 4.36; 95% CI: 1.74 - 10.93) were significantly associated with 30-day all-cause hospital readmission.

**Conclusions:**

More than a quarter of patients hospitalized for severe CAP were readmitted within 30 days, and nearly two-thirds were readmitted within two weeks. Patients with severe CAP who were admitted with pulmonary disease, multiple comorbidities, discharged with clinical instability, and developed severe CAP complications were more likely to be readmitted to the hospital. Thus, the provision of optimized in-hospital care, clear discharge planning, post-discharge follow-up, patient education, medication reconciliation, and vaccination can reduce readmission rates.

**Supplementary information:**

The online version contains supplementary material available at 10.1186/s12879-026-13064-5.

## Background

Hospital readmissions are a frequent complication of community acquired pneumonia (CAP) [1–5], and approximately one-fourth (28.6%) of patients hospitalized for CAP are readmitted within 30 days [6]. Hospital readmission following CAP is linked to significant adverse clinical events such as in-hospital death, prolonged length of hospital stay, admission to the intensive care unit, and use of mechanical ventilation. Readmission after CAP is associated with serious clinical outcomes, including increased in-hospital mortality, longer hospital stays, ICU admissions, and the need for mechanical ventilation [7, 8]. Furthermore, it may lead to an increased treatment burden, medical resource use and costs, and lower patient satisfaction [7–9]. In a few high-income settings (such as the US), there is a slight and modest declining trend in 30-day all-cause readmission after CAP [10]. However, in many settings, particularly those with fewer resources, there are insufficient data to estimate current trends.

Despite there being no single a single global estimate for 30-day CAP readmission due to variability by country, healthcare setting, and patient characteristics, studies conducted in high-income countries reported that reasonable estimates range from 8.4% to 32% [3, 5–7, 11–16]. In Africa, a study conducted in South Africa found a 30-day CAP readmission rate of 10.5% [14]. Evidence has shown that a number of factors such as “instability” on hospital discharge [17], host factors (age, financial barriers) [6], CAP-related factors (severity of CAP) [18], treatment factors (inappropriate antibiotic therapy, medication adherence), and the presence of certain comorbidities (chronic obstructive pulmonary disease, heart failure) [3, 11, 15, 18, 19] are associated with increased readmission.

In low-resource settings such as Ethiopia, where infection control is poor [20] and the burden of antimicrobial resistance high [21], early hospital readmission may have a devastating impact on healthcare services. In addition, previous studies have shown that early hospital readmission has significant clinical and economic impacts in low- and middle-income countries, including Ethiopia [22, 23]. Given the adverse clinical outcomes and economic impact of early CAP hospital readmission [7, 8, 24], reducing readmission is crucial in the management of CAP. To develop effective interventions that can decrease readmission rates of severe community-acquired pneumonia (SCAP), it is essential to determine its magnitude and identify factors for increased re-hospitalization. Identifying patients at high risk of short-term rehospitalization after discharge for SCAP is urgently needed to ensure that healthcare attention is given to those with highest needs. Factors associated with short-term rehospitalization have been previously investigated. However, these studies have some limitations because of the failure to use a rigorous definition of CAP, variation in the study population (e.g., inclusion of only elderly patients) [3, 4, 11], retrospective designs based on administrative records [11], and failure to explore the distinction between CAP and healthcare-associated pneumonia on readmission. In addition, most of these studies were conducted in high-income countries, and there is insufficient data from the low-income countries. In Ethiopia, previous studies on factors associated with readmission have focused on medical conditions such as heart failure and diabetes mellitus [25, 26]. According to the literature search, no studies have been conducted on the extent of 30-day readmission and its risk factors among patients with SCAP in Ethiopia, including in the study setting. In this study, SCAP was defined according to the 2007 the Infectious Diseases Society of America (IDSA) [27]. This study hypothesized that patients hospitalized with SCAP have a measurable prevalence of 30-day all-cause hospital readmission and that specific demographic and clinical factors are significantly associated with the likelihood of readmission. This study aimed to determine the prevalence and factors associated with 30-day all-cause readmission after SCAP hospitalization at UOGCSH, Northwest Ethiopia.

## Methods and materials

### Study design, setting and period

A cross-sectional study was conducted prospectively among patients with SCAP who were admitted to the medical wards of the University of Gondar Comprehensive Specialized Hospital (UOGCSH) between July 1, 2023, and June 30, 2024. The University of Gondar Comprehensive Specialized Hospital (UOGCSH) is a tertiary teaching hospital located 738 km away from the capital city, Addis Ababa, Ethiopia. It serves more than 10 million people in Northwest Ethiopia. The facility has nearly more than 900 beds in different departments, including internal medicine, emergency, pediatrics, ambulatory, surgery, oncology, gynecology, psychiatry, HIV care, dermatology, and orthopedics. Hospitalized patients with SCAP are managed in the internal medicine ward.

### Eligibility criteria

The study included adult patients (aged ≥ 18 years) diagnosed with SCAP who received treatment during their index hospitalization, were admitted to the medical ward between July 1, 2023, and June 30, 2024, were discharged alive with a diagnosis of SCAP, and provided written informed consent. Patients were excluded if they had a history of hospitalization within 30 days, stayed in the hospital for less than one day, were transferred to another acute care facility, were discharged against medical advice, or had a scheduled hospital admission.

### Sample size determination and sampling procedure

The minimum number of samples required for the study was determined using the single population proportion formula, considering the following assumptions:

$${{\mathrm{n}}_0} = \frac{{{{\mathrm{Z}}^2}p\left( {1 - P} \right)}}{{{{\mathrm{d}}^2}}}$$

$${{\mathrm{n}}_0} = \frac{{{{(1.96)}^2}*0.119*\left( {1 - 0.119} \right)}}{{{{\left( {0.05} \right)}^2}}}$$

n_0_ = 161.1

where n_0_ = minimum sample size required for the study; Z = standard normal distribution (Z = 1.96) with a confidence interval (CI) of 95% and ⍺ = 0.05; *p* = expected prevalence of 30-day all-cause readmission following hospitalization for CAP (*p* = 11.9%) [7]; and W = Absolute precision (W) = 0.05. Then, adding 10% (161 × 0.1 = 16) of the non-response rate, the total sample size for the study was (161+16) = 177. Once the required sample size was achieved, study participants who fulfilled the inclusion criteria were selected using consecutive sampling.

### Data collection procedures

Data were collected by using a pretested data abstraction format which was prepared by reviewing previously published similar studies [1–6, 11, 16, 18, 28] (Supplementary file), and it was collected by four trained nurses. Index hospitalization characteristics of the patients, including data regarding socio-demographic variables, vital signs, behavioral factors (smoking and alcohol use), clinical presentation on admission, comorbidities, X-ray and laboratory findings, CURB-65 score (confusion, urea, respiratory rate, blood pressure, age ≥ 65 years), antibiotics used during the hospital course, and measures of clinical instability were collected from medical chart review and patient interviews. After the patients’ baseline characteristics were collected, from their date of discharge until 30 days post-discharge, patients were followed up for the occurrence of readmission. Information on readmission, overall time to readmission, and reasons for readmission was collected by re-review of index hospital medical records up to 30 days after the initial discharge. If there was no evidence that the patient had been readmitted to the hospital, contact was made with them via telephone to confirm their re-hospitalization at other healthcare facilities within 30 days. In addition, during post-discharge, when the patient was not reachable for any reason or had changed contact information, their relatives were contacted through an alternative telephone as a secondary option.

In the study between index hospitalization discharge and readmission, additional care was provided to patients through telephone calls. For instance, patients were counseled to adhere to their medications for the treatment of both SCAP and comorbidities. Patients were counseled on self-management practices for CAP and their comorbid conditions at home. In addition, they were followed up for improvement in their clinical status and medication adherence. Moreover, after being readmitted to the hospital, patients received care that was better than the standard CAP management, including identification and addressing of the causes of readmission, confirmatory diagnosis, reviewing and adjustment of antibiotic therapy, and evaluation and management of complications and comorbidities.

### Data quality control technique

To assure the completeness of the data abstraction format, a pre-test was conducted among 18 patients with SCAP (10% of the sample size), and appropriate modifications were made to the format. The pretest was conducted at the UOGCSH emergency medical inpatient ward, and the data collected for the pretest were excluded from the analysis. Furthermore, during the data collection period, with the help of two supervisors, supervision was held regularly, and the collected data were checked daily for completeness and consistency. Before conducting the actual data collection, the data collectors were trained on the purpose of the study and the data collection process.

### Data entry and statistical analysis

Data were cleaned, coded, entered, and analyzed using the Statistical Package for Social Sciences (SPSS) version 25. The Kolmogorov-Smirnov test was used to check for normality. Continuous variables were expressed as mean ± standard deviation when normally distributed or median (interquartile range, IQR) when not normally distributed. Categorical variables were summarized as frequencies (percentages). Multicollinearity was checked using the variance inflation factor (VIF). Thirty (30)-day all-cause hospital readmission was used as a dependent variable, while patient characteristics, including demographic, behavioral factors, clinical presentation, vital signs, comorbid conditions, physical examination, X-ray findings, laboratory values, oxygen saturation, CURB-65 score (confusion, urea, respiratory rate, blood pressure, age ≥ 65 years), treatment (in-hospital antibiotic therapy), index hospital admission course (ICU admission), index hospital stay, and measures of clinical instability before hospital discharge were used as independent variables. Independent variables with *p*-values < 0.05 in the univariable binary logistic regression model were chosen for the multivariable regression model. Multivariable binary logistic regression analysis was used to identify factors associated with all-cause 30-day readmission following SCAP hospital discharge. Results of the regression were presented as crude odds ratio (COR) and adjusted odds ratios (AOR) with 95% CI. A two-tailed *p*-value < 0.05 was considered statistically significant to declare the association. The Hosmer–Lemeshow test (Chi-square (χ2) = 9.564, *p*-value = 0.297) was used to evaluate the fitness of the logistic regression model.

### Study variables and operational definitions

30-day all-cause hospital readmission: Defined as ‘hospitalization for any reason within 30 days following discharge after the index SCAP hospitalization, excluding scheduled hospital admission and unavoidable emergencies such as a car accident or combat injury.

Severe community-acquired pneumonia (SCAP): Defined according to the Infectious Diseases Society of America (IDSA) 2007 criteria [27], as follows: (1) having one or more of the major criteria (invasive mechanical ventilation or septic shock with a need for vasopressors); (2) having three or more of the minor criteria (respiratory rate ≥ 30 breaths/min, PaO_2_/FiO_2_ ≤ 250 mmHg, multilobar infiltrates, confusion/disorientation, blood urea nitrogen (BUN) ≥20 mg/dL, WBC count < 4000 cells/mm^3^, platelet count < 100,000 cells/mm^3^, body temperature < 36 °C, hypotension requiring aggressive fluid resuscitation).

Clinical instability at hospital discharge: Measures of clinical instability criteria within 24 h before hospital discharge were defined using the 2007 IDSA/ATS [27].

Hospital readmission was classified as SCAP-related, SCAP-unrelated, or a combination of SCAP- and comorbidity-related.

- SCAP-related hospital readmission: Defined if pneumonia was an immediate or underlying cause of the readmission or if it played a major role in the readmission [28].
- SCAP-unrelated hospital readmission: Defined if the clinical data suggested an alternative reason for the readmission. Examples of these diagnoses include heart failure, hypertension, and kidney disease [28].
- combination of SCAP- and comorbidity-related readmission: If, the primary reason for readmission could not be accurately determined as in the case of patients with exacerbations of chronic lung disease, they were categorized as a combination of SCAP- and comorbidity-related readmission [28].

## Result

### Socio-demographic and behavioral characteristics of patients with SCAP

During the study period. 476 patients with SCAP were admitted and discharged alive from the medical ward of UOGCSH. Of these, 177 consecutive patients with SCAP were included, and 299 patients who did not meet the inclusion criteria were excluded from the study; 126 had scheduled hospital admission, 76 had a history of hospitalization within 3 months, 42 didn’t provide written informed consent, 32 were transferred to another acute-care facility, and 23 left against medical advice.

Among 177 patients with SCAP included in the study, nearly two-thirds (67.8%) were male and 55.4% were from rural areas. Their median age was 45 (IQR: 28–62.5) and approximately half (51.4%) were married (Table 1).

**Table 1:** Socio-demographic and behavioral characteristics of patients with SCAP at UOGCSH, Northwest, Ethiopia, 2024 (*N* = 177)

Variable	Category	Frequency (%)
Age	18–35 years	71 (40.1)
	36–49 years	30 (16.9)
	50–64 years	24 (13.6)
	≥65 years	52 (29.4)
Sex	Male	120 (67.8)
	Female	57 (32.2)
Marital status	Married	91 (51.4)
	Single	38 (21.5)
	Divorced	25 (14.1)
	Widowed	23 (13.0)
Level of education	Not able to read and write	69 (39.0)
	Able to read and write	16 (9.0)
	Primary	24 (13.6)
	Secondary	40 (22.6)
	College and above	28 (15.8)
Occupation	Housewife	48 (27.1)
	Farmer	63 (35.6)
	Government employee	25 (14.1)
	Daily laborer	22 (12.4)
	Student	12 (6.8)
	Private organization employed	7 (4.0)
Residence	Urban	79 (44.6)
	Rural	98 (55.4)
Cigarette smoking	No	160 (90.4)
	Yes	17 (9.6)
		160 (90.4)
Alcohol consumption	No	134 (75.7)
	Yes	43 (24.3)
	No	134 (75.7)

%; percent; SCAP: Severe Community Acquired Pneumonia; UOGCSH: University of Gondar Compressive Specialized Hospital

### Clinical and treatment characteristics of patients with SCAP at index admission and on discharge

The mean (±SD) pulse rate of the study participants was 97.64 (±15.97) beats/minute while their median (IQR) respiratory rate was 26 (IQR: 22.0–28.0) breaths/minute. More than one-fourth of the study participants were admitted with oxygen desaturation (33.3%), leukocytosis (28.2%), parapneumonic effusion (28.8%), and multilobar infiltrates (28.2%). During the index hospital course, nearly one-quarter (26.6%) of the patients were admitted to the intensive care unit (ICU), and their median hospital stay was 13 (IQR: 11–17) days. Ceftriaxone (90.4%) was the most frequently used antibiotic for treatment of SCAP followed by azithromycin (52.5%) and vancomycin (33.3%). Prior to hospital discharge, more than one-quarter (37.3%) of the patients were discharged with ≥1 physical examination finding showing clinical instability at discharge (Table 2).

**Table 2:** Clinical and treatment characteristics of patients with SCAP at UOGCSH, Northwest, Ethiopia, 2024 (*N* = 177)

Variable	Category	Mean (±SD) or Median (IQR)	Frequency (%)
Vital signs			
	SBP (mmHg)	110 (100–130) ^##^	NA
	DBP (mmHg)	75 (60–90) ^##^	NA
	Temperature (^0^C)	37.3 (36.6–38.0) ^##^	NA
	Pulse rate (beats/minute)	97.64 (±15.97)^#^	NA
	Respiratory rate (breath/minute)	26 (22.0–28.0) ^##^	NA
Oxygen saturation		92 (81.0–95.0) ^##^	NA
Laboratory values			
	White blood count (10^3^/mm^3^)	6.9 (5.25–10.3) ^##^	NA
	Neutrophil (%)	71.6 (57.95–82) ^##^	NA
	Platelet count	213 (155–283) ^##^	NA
	Hemoglobin (g/dl)	13.5 (10.0–14.2) ^##^	NA
	Serum sodium (mmol/l)	136 (132.0–139.0) ^##^	NA
	Serum potassium (mmol/l)	3.83 (3.40–4.21) ^##^	NA
	Serum creatinine (mg/dl)	0.74 (0.54–1.10) ^##^	NA
	Blood urea level (mg/dl)	28 (21–40) ^##^	NA
Complication of SCAP			
	Empyema	NA	44 (24.9)
	Parapneumonic effusion	NA	51 (28.8)
	Respiratory failure	NA	52 (29.4)
CURB-65 score		3 (3,4) ^##^	NA
CURB-65 score category			
	3	NA	118 (66.7)
	4	NA	33 (18.6)
	5	NA	26 (14.7)
Antibiotics used for treatment of SCAP			
	Ceftriaxone	NA	160 (90.4)
	Azithromycin	NA	93 (52.5)
	Vancomycin	NA	59 (33.3)
	Clindamycin	NA	47 (26.6)
	Doxycycline	NA	37 (20.9)
	Ciprofloxacin	NA	33 (18.6)
	Cefepime	NA	12 (6.8)
Physical examination finding indicating instability on discharge			
	Respiratory rate > 24 (breath/minute)	NA	22 (12.4)
	Pulse rate > 100 (beats/minute)	NA	19 (10.7)
	SBP < 90	NA	14 (7.9)
	Temperature > 37.8	NA	20 (11.3)
	Inability to maintain oral intake	NA	17 (9.6)
Presence of ≥ 1 clinical instability at discharge			
	No	NA	111 (62.7)
	Yes	NA	66 (37.3)

NA: Not applicable; %; percent; SD: Standard Deviation: IQR: Inter Quartile Range; SCAP: Severe Community Acquired Pneumonia; SBP: Systolic Blood Pressure; DBP: Diastolic Blood Pressure; CURB-65: Confusion, Urea, Respiratory Rate, Blood Pressure, Age ≥ 65 years; UOGCSH: University of Gondar Compressive Specialized Hospital; ^#^: mean (±SD); ^##^: median (IQR)

**Fig. 1:**
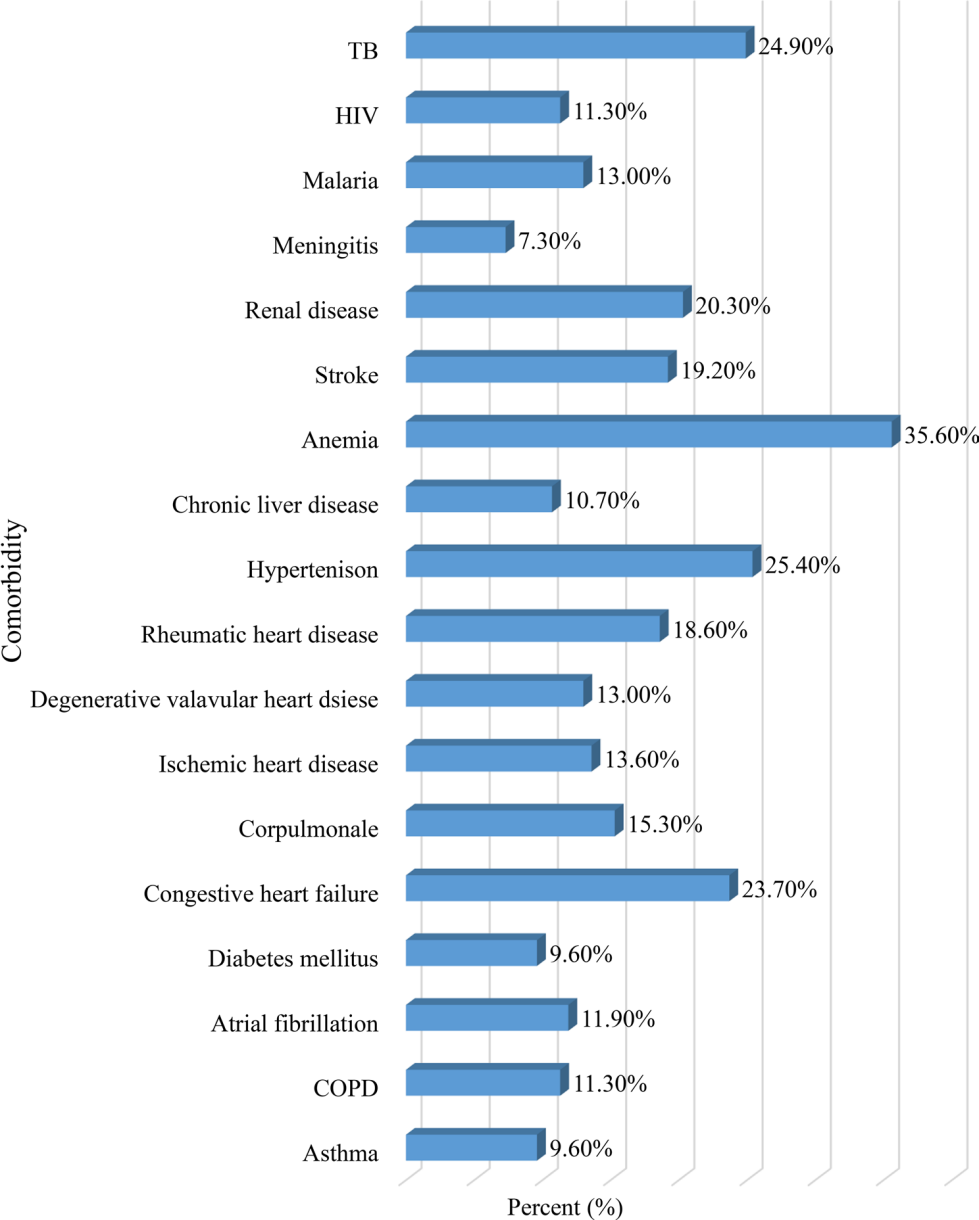
Comorbidities among patients with SCAP at UOGCSH, Northwest, Ethiopia, 2024 (*N* = 177). SCAP: severe community acquired Pneumonia; COPD: chronic obstructive pulmonary Disease; HIV: human immunodeficiency Virus; TB: Tuberculosis; UOGCSH: University of Gondar Compressive Specialized Hospital

**Fig. 2:**
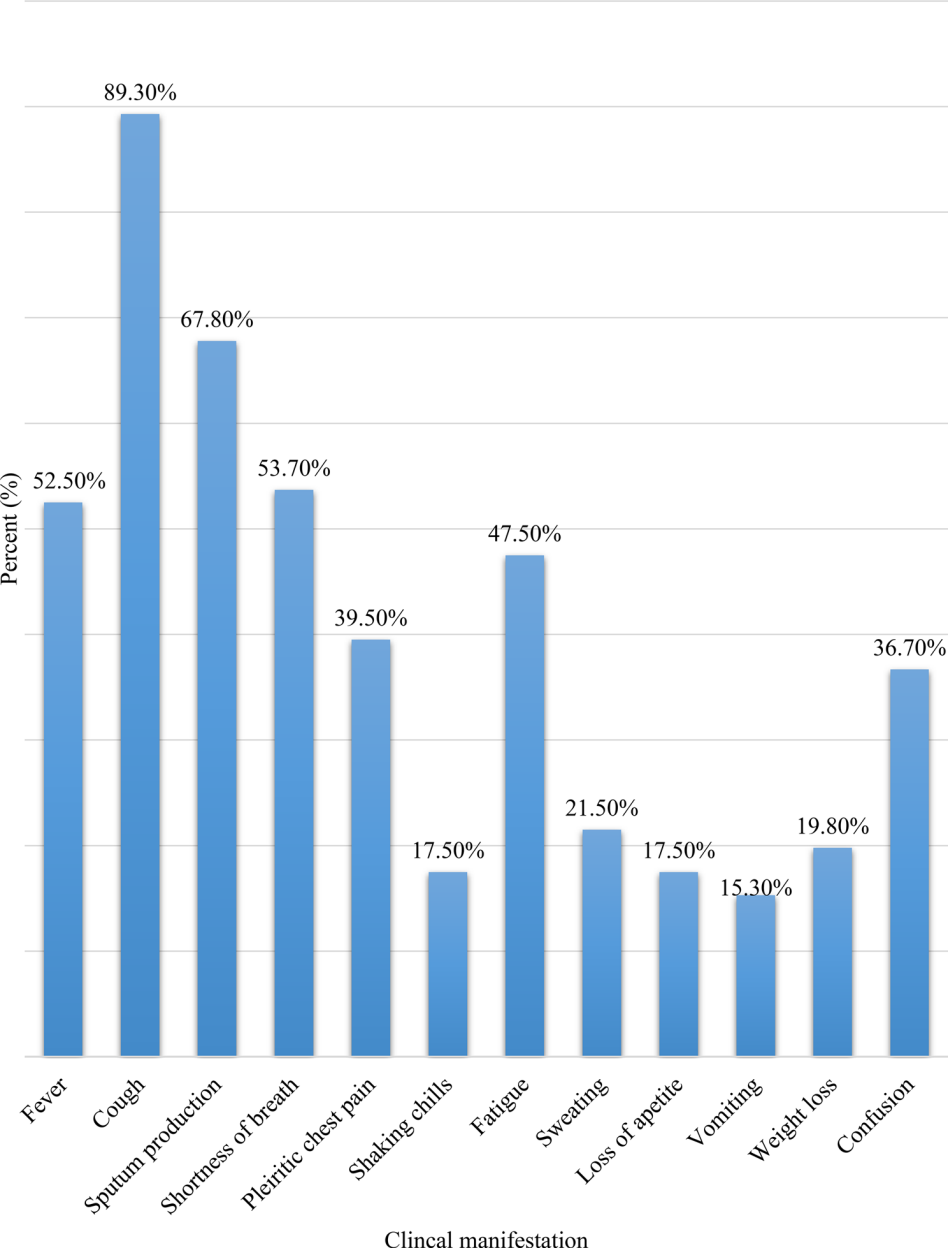
Clinical presentation of patients with SCAP at UOGCSH, Northwest, Ethiopia, 2024 (*N* = 177). SCAP: Severe Community Acquired Pneumonia; UOGCSH: University of Gondar Compressive Specialized Hospital

**Fig. 3:**
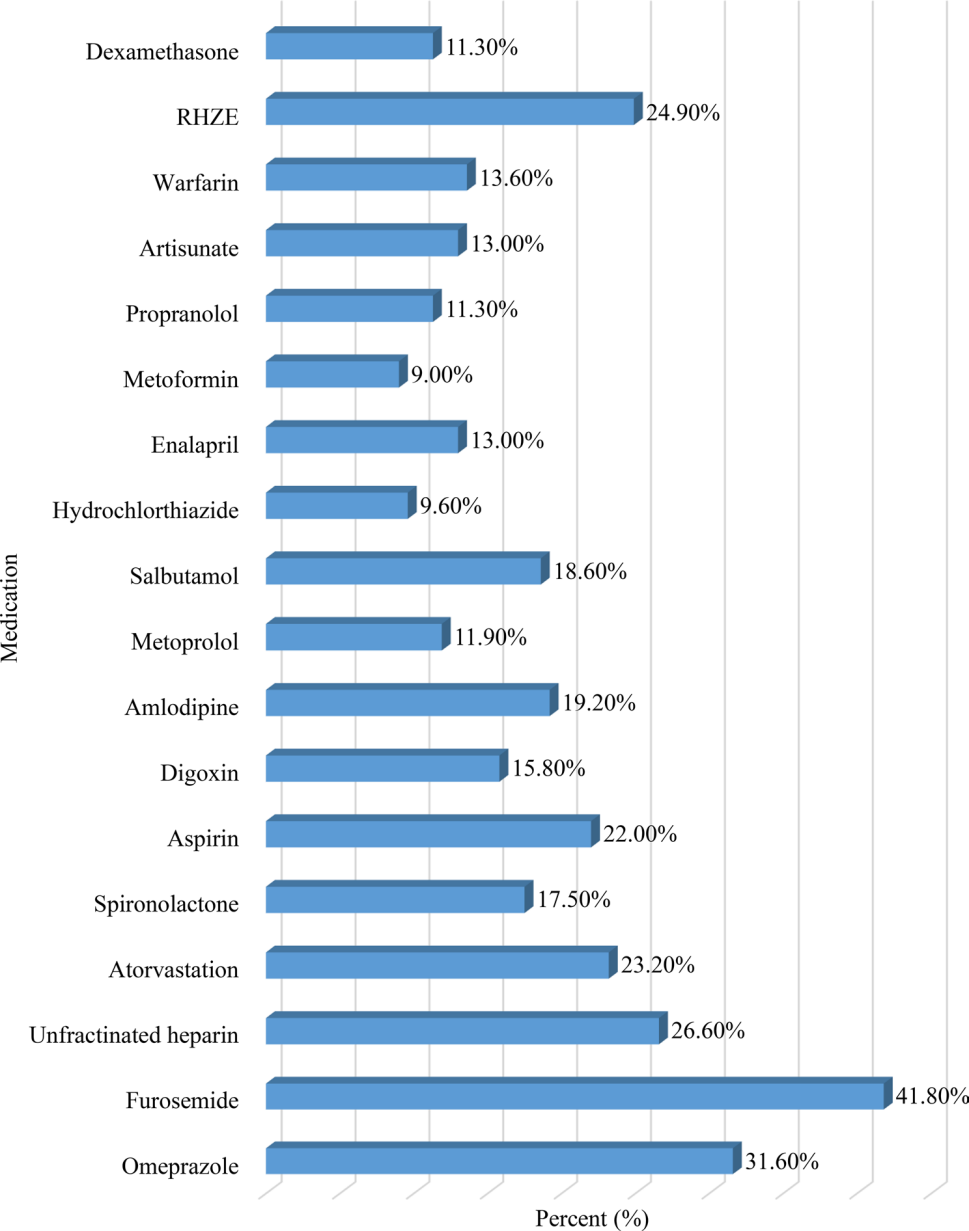
Medications for treatment of comorbidities among patients with SCAP at UOGCSH, Northwest, Ethiopia, 2024 (*N* = 177). SCAP: Severe Community Acquired Pneumonia; UOGCSH: University of Gondar Compressive Specialized Hospital; RHZE: Rifampicin-Isoniazid-Pyrazinamide-Ethambutol

Approximately half (51.4%) of the study participants had ≥ 3 comorbidities; anemia (35.6%), hypertension (25.4%), and tuberculosis (24.9%) were the most frequently diagnosed comorbidities (Fig. 1). The majority (89.3%) of the patients presented with cough, followed by sputum production (67.8%) and shortness of breath (53.7%) (Fig. 2). Furthermore, furosemide (41.8%), omeprazole (31.6%), unfractionated heparin (26.6%), and rifampicin-isoniazid-pyrazinamide-ethambutol (24.9%) were the most frequently prescribed medications for the treatment of comorbidities (Fig. 3).

### Prevalence and causes of 30-day all cause hospital readmission following SCAP hospital discharge

The prevalence of 30-day all-cause hospital readmission among patients with SCAP was 29.9% (95% CI: 23.3–37.3), with 71.7% of readmissions occurring within the first two weeks. The median (IQR) time to hospital readmission was 13 days (IQR: 11.5–15.5 days). Overall, 45.3% of readmissions were comorbidity-related, 33.9% were SCAP-related, and 20.7% were a combination of both comorbidity- and SCAP-related. Among the 53 (29.9%) patients who were readmitted to the hospital, eight (15.06%) died in the hospital.

### Factors associated with 30-day all cause hospital readmission following SCAP hospital discharge

In the multivariable logistic regression model, COPD increased the predicted probability of 30-day readmission from 29.9% to 65.8%, corresponding to an absolute increase of 35.9% points (AOR = 4.51; 95% CI: 1.19–17.15). The presence of fever increased the predicted probability from 29.9% to 57.1%, an absolute increase of 27.2% points (AOR = 3.12; 95% CI: 1.26–7.73). Admission with 3 or more comorbidities was associated with an increase in predicted readmission probability to 53.3%, representing an absolute increase of 23.4% points (AOR = 2.68; 95% CI: 1.11–6.50). Patients discharged with at least one clinical instability factor had a predicted probability of readmission of 52.0%, an absolute increase of 22.1% points compared with clinically stable patients (AOR = 2.54; 95% CI: 1.06–6.13). Among the complications of SCAP, parapneumonic effusion increased the predicted probability of readmission to 57.2%, an absolute increase of 27.3% points (AOR = 3.13; 95% CI: 1.26–7.77), while respiratory failure increased the predicted probability to 65.0%, corresponding to an absolute increase of 35.1% points (AOR = 4.36; 95% CI: 1.74–10.93) (Table 3).

**Table 3:** Factors associated with 30-day all-cause readmission of patients with SCAP at UOGCSH, Northwest Ethiopia (*N* = 177)

Variable	Category	30-day all cause readmission n		COR (95%CI)	AOR (95%CI)	P-value
		Yes	No			
Chronic Obstructive Pulmonary Disease						
	No	40	117	Ref	Ref	
	Yes	13	7	5.43 (2.03,14.57)	4.51 (1.19,17.15)	0.027*
Fever						
	No	12	72	Ref	Ref	
	Yes	41	52	4.73 (2.27,9.87)	3.12 (1.26,7.73)	0.014*
Confusion						
	No	19	93	Ref	Ref	
	Yes	34	31	5.37 (2.68,10.74)	1.35 (0.53,3.45)	0.527
Respiratory failure-complications of SCAP						
	No	20	105	Ref	Ref	
	Yes	33	19	9.12 (4.35,19.11)	4.36 (1.74,10.93)	0.002*
Parapneumonic effusion-complications of SCAP						
	No	23	103	Ref	Ref	
	Yes	30	21	6.39 (3.12,13.12)	3.13 (1.26,7.77)	0.014*
Presence of ≥ 1 clinical instability						
	No	25	86	Ref	Ref	
	Yes	28	38	2.54 (1.31,4.91)	2.54 (1.06,6.13)	0.037*
Number of comorbidity						
	0–2	12	74	Ref	Ref	
	≥3	41	50	5.06 (2.42,10.56)	2.68 (1.11,6.50)	0.029*

n (%): Frequency (percentage); SCAP: Severe Community-Acquired Pneumonia; UOGCSH: University of Gondar Compressive Specialized Hospital; Ref: Reference category; COR: Crude Odd Ratio; AOR: Adjusted Odd Ratio; CI: Confidence Interval. *statistically significant at *p*-value < 0.05

## Discussion

Despite efforts to reduce readmission rates for patients with CAP, including in developed countries, only a modest decline has been achieved, and factors such as an aging population and multiple complex comorbidities continue to drive persistent readmissions. Determining the extent and identifying the risk factors of hospital readmission is crucial for developing interventions to reduce SCAP readmission rates. This study showed that the prevalence of 30-day all-cause hospital readmission among patients with SCAP was 29.9% (95% CI: 23.3 to 37.3), and the majority (71.7%) of patients were readmitted within 2 weeks. Admission with COPD, fever, ≥3 comorbidities, clinical instability at discharge, and SCAP complications such as parapneumonic effusion and respiratory failure were significantly associated with 30-day all-cause hospital readmission.

The readmission rate observed in this study was in line with the results reported in previous studies conducted in Spain (27%) [11], Germany (28.6%) [6], and the United Kingdom (26%) [5]. However, it was higher than the rates reported in studies conducted in Korea (8.4%) [18], South Africa (10.5%) [14], Danish Hospitals (16.0%) [29], and Vietnam (11.9%) [7]. This discrepancy might be explained by differences in the study population, the definition of pneumonia, study designs, and data collection and analysis. For instance, a previous study in Spain, which was conducted to determine the factors associated with readmission for community-onset pneumonia, included only older adults [11]. Furthermore, this study showed that the majority (71.7%) of patients were readmitted to the hospital ≤ 14 days after the initial hospital discharge. This was corresponded to a previous study in the United Kingdom, which revealed that 66% of readmissions occurred within 14 days of discharge [5].

Comorbidity-related readmission (45.3%) was the most frequent reason for 30-day all-cause hospital readmissions, and congestive heart failure (9%), stroke (7.3%), and renal disease (6.8%) were the most frequent comorbid conditions. Other studies have also shown that the most common reason for readmission is new or worsening comorbidities unrelated to pneumonia, mainly cardiovascular and pulmonary diseases [2, 15, 18].

In this study COPD, fever, and 3 or more comorbidities increased the predicted probability of 30-day readmission from 29.9% to 65.8%, 57.1%, and 53.3%, respectively. These results are consistent with previous studies showing that a high comorbidity burden increases the risk of in patients with CAP [1, 4, 28, 29]. For instance, a Danish retrospective study found that having ≥ 2 comorbidities independently increased the risk of 30-day all-cause readmission after CAP [29]. A cross-sectional study in Spain reported that chronic respiratory failure was one of the factors independently associated with 30-day readmission [3]. These associations could result in an increased risk of complications after hospital discharge and a high rate of early hospital readmissions. In the current study, SCAP complications specifically, parapneumonic effusion and respiratory failure, increased the predicted probability of readmission to 57.2% and 65.0%, respectively. In this regard, evidence has documented that the occurrence of CAP complications in an individual patient during hospitalization may lead to further CAP-related worse clinical outcomes, such as mortality and an increased risk of hospital readmission [30]. Furthermore, patients discharged with at least one clinical instability factor had a predicted probability of readmission of 52.0% compared with clinically stable patients. In most studies, ≥1 clinical instability prior to hospital discharge was reported to be an important factor associated with pneumonia-related hospital readmission [17, 18]. Implementing strategies such as optimized in-hospital care, clear discharge planning, post-discharge follow-up, patient education, medication reconciliation, and vaccination can reduce readmission rates. By identifying the extent and modifiable risk factors of readmission, these findings can guide institutions and providers in developing interventions to reduce early hospital readmissions for patients with SCAP. Furthermore, it will provide baseline data for future studies conducted in low-income countries, where there is a scarcity of data regarding the prevalence of 30-day all-cause hospital readmission and its associated factors among patients with SCAP.

Despite its prospective nature, this study had some limitations. First, as it was conducted in a single-center health facility with a small sample size, the generalizability of the results might be limited. Second, as it was a cross-sectional study, it could not show the cause-effect relationship between 30-day all-cause readmission and significant associated factors. Third, it lacks the evaluation of in-hospital and discharge antibiotic use appropriateness, which may influence the patient’s condition after hospital discharge.

## Conclusions

More than a quarter of patients hospitalized for SCAP were readmitted within 30 days, and nearly two-thirds of these patients were readmitted within 2 weeks. Patients with SCAP who were admitted with COPD, fever, three or more comorbidities, discharged with one or more clinical instability, and developed SCAP complications, specifically parapneumonic effusion and respiratory failure, were more likely to be readmitted to the hospital. Thus, optimizing in-hospital care, clear discharge planning, post-discharge follow-up, patient education, medication reconciliation, and vaccination can reduce readmission rates.

## Electronic supplementary material

Below is the link to the electronic supplementary material.

Supplementary Material 1

## Data Availability

The datasets used/or analyzed during the current study is available from the corresponding author on reasonable request.

## Abbreviations

AOR: Adjusted Odd Ratio
CI: Confidence Interval
COPD: Chronic Obstructive Pulmonary Disease
COR: Crude Odd Ratio
CURB: Confusion, Urea, Respiratory Rate, Blood Pressure
DBP: Diastolic Blood Pressure
HIV: Human Immunodeficiency Virus
ICU: Inter-Critical Unit
IQR: Inter Quartile Range
SBP: Systolic Blood Pressure
SCAP: Sever Community Acquired Pneumonia
SD: Standard Deviation
SPSS: Statistical Package for Social Sciences
TB: Tuberculosis
UOGCSH: University of Gondar Comprehensive Specialized Hospital
