# Supplementary material for: Thirty-day hospital readmission and its determinants among patients with severe community-acquired pneumonia: a prospective cross-sectional study in Northwest Ethiopia
Source: BMC Infect Dis. 2026 Mar 17;26:827. doi: 10.1186/s12879-026-13064-5 (PMC13107582; doi:10.1186/s12879-026-13064-5)
Supplement: Supplementary file 1 — Supplementary Material 1 [file 12879_2026_13064_MOESM1_ESM.docx]

**Data collection tool for 30-day hospital readmission and its associated factors among patients hospitalized with severe community-acquired pneumonia at University of Gondar Comprehensive Specialized Hospital: A prospective cross-sectional study**

**Part I: Socio-demographic and behavioral characteristics of patients with severe community-acquired pneumonia (SCAP)**

1. Sex Male Female
2. Age (years): _________________
3. Residence Urban Rural
4. Marital status Married Single Divorced Widowed
5. Level of education Not able to read and write

Able to read and write

Primary

Secondary

College and above

1. Occupation Housewife

Farmer

Government employee

Daily laborer

Student

Private organization employee

1. Current cigarette smoking status Yes No
2. Current alcohol drinking status Yes No

**Part II: Clinical and treatment characteristics of patients with SCAP at index admission and on discharge**

1. Vital signs SBP (mmHg): ______ DBP (mmHg): ______ Temperature (^0^C): _______

Pulse rate (beats/minute): ______ Respiratory rate (breath/minute): ______

1. Oxygen saturation (%): _____________
2. Clinical manifestations

Cough Fever Sputum production

Shaking chills Sweating Vomiting

Loss of appetite Shortness of breath Weight loss

Confusion Fatigue Pleuritic chest pain

1. Comorbidity

Tuberculosis Anemia Hypertension

Meningitis Renal disease Stroke

Diabetes mellitus Asthma Corpulmonale

Atrial fibrillation Malaria Rheumatic heart disease

Degenerative valvular heart disease Ischemic heart disease

Congestive heart failure Chronic liver disease

Chronic obstructive pulmonary disease Human immunodeficiency virus

1. Laboratory values White blood count (10^3^/mm^3^): _____ Neutrophil (%): __________

Platelet count: _________ Hemoglobin (g/dl): ________

Serum sodium (mmol/l): __________Serum potassium (mmol/l): ______

Serum creatinine (mg/dl): _________ Blood urea level (mg/dl): _________

1. Complication of SCAP

Empyema Parapneumonic effusion Respiratory failure Multi-lobar infiltrate

1. CURB-65 (confusion, urea, respiratory rate, blood pressure, age ≥ 65 years) score: ______
2. Antibiotics used for treatment of SCAP

Ceftriaxone Azithromycin Vancomycin Clindamycin Doxycycline Ciprofloxacin Cefepime

1. Medications used for treatment of comorbidities

Furosemide Omeprazole Unfractionated heparin

Dexamethasone Salbutamol Warfarin

Artisunate Propranolol Metoformin

Enalapril Hydrchlorthiazide Metoprolol

Amlodipine Digoxin Aspirin

Spironolactone Atorvastatin

Rifampincin-isoniazid-pyrazinamide-ethambutol

1. Length of hospital stay (days): __________
2. Admission to inter-critical unit (ICU) Yes No
3. Presence of ≥ 1 clinical instability at discharge Yes No
4. Physical examination finding indicating instability on discharge

Respiratory rate > 24 (breath/minute) Pulse rate >100 (beats/minute)

SBP < 90 (mmHg) Temperature > 37.8^0^C

Inability to maintain oral intake

**Part III: Assessment of outcome variable (i.e 30-day all cause hospital readmission)**

**Part III A: Through re-review of index hospital medical records:** to confirm patients were readmitted to University of Gondar Comprehensive Specialized Hospital (UOGCSH)

1. Presence of 30-day all cause hospital readmission to UOGCSH following SCAP hospital discharge

Yes No

1. Reason for hospital readmission

CAP related Comorbidity related Both CAP and comorbidity related

1. Date of hospital readmission (days): __________

**Part III B: Through phone call patient interview:** when there was no evidence that the patient had readmitted to UOGCSH and to confirm their re-hospitalization to other healthcare facilities within 30-days.

1. Did you readmitted to a healthcare facility other than UOCGSH with in the past 30 days?

Yes No

1. If it was yes what was your reason for readmission?

CAP related Comorbidity related Both CAP and comorbidity related

1. Date of hospital readmission: ________________________
